# Assessing Oxidative Stress in Tumors by Measuring the Rate of Hyperpolarized [1-^13^C]Dehydroascorbic Acid Reduction Using ^13^C Magnetic Resonance Spectroscopy*

**DOI:** 10.1074/jbc.M116.761536

**Published:** 2016-12-19

**Authors:** Kerstin N. Timm, De-En Hu, Michael Williams, Alan J. Wright, Mikko I. Kettunen, Brett W. C. Kennedy, Timothy J. Larkin, Piotr Dzien, Irene Marco-Rius, Sarah E. Bohndiek, Kevin M. Brindle

**Affiliations:** From the ‡ Department of Biochemistry,; the § Cancer Research UK Cambridge Institute, and; the ¶ Department of Physics, University of Cambridge, Cambridge CB2 0RE, United Kingdom

**Keywords:** glutathione peroxidase, in vivo imaging, oxidative stress, pentose phosphate pathway (PPP), tumor metabolism, 13C, dehydroascorbic acid, glutathione, hyperpolarization

## Abstract

Rapid cancer cell proliferation promotes the production of reducing equivalents, which counteract the effects of relatively high levels of reactive oxygen species. Reactive oxygen species levels increase in response to chemotherapy and cell death, whereas an increase in antioxidant capacity can confer resistance to chemotherapy and is associated with an aggressive tumor phenotype. The pentose phosphate pathway is a major site of NADPH production in the cell, which is used to maintain the main intracellular antioxidant, glutathione, in its reduced state. Previous studies have shown that the rate of hyperpolarized [1-^13^C]dehydroascorbic acid (DHA) reduction, which can be measured *in vivo* using non-invasive ^13^C magnetic resonance spectroscopic imaging, is increased in tumors and that this is correlated with the levels of reduced glutathione. We show here that the rate of hyperpolarized [1-^13^C]DHA reduction is increased in tumors that have been oxidatively prestressed by depleting the glutathione pool by buthionine sulfoximine treatment. This increase was associated with a corresponding increase in pentose phosphate pathway flux, assessed using ^13^C-labeled glucose, and an increase in glutaredoxin activity, which catalyzes the glutathione-dependent reduction of DHA. These results show that the rate of DHA reduction depends not only on the level of reduced glutathione, but also on the rate of NADPH production, contradicting the conclusions of some previous studies. Hyperpolarized [1-^13^C]DHA can be used, therefore, to assess the capacity of tumor cells to resist oxidative stress *in vivo*. However, DHA administration resulted in transient respiratory arrest and cardiac depression, which may prevent translation to the clinic.

## Introduction

**FIGURE 1.**
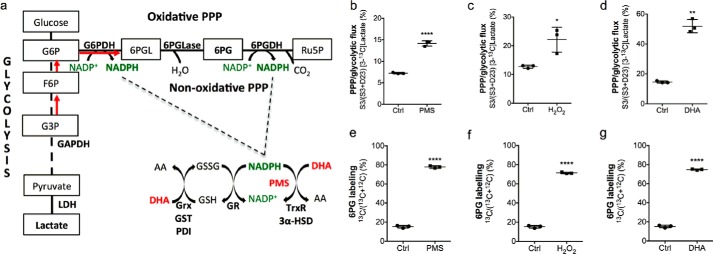
**PPP flux in oxidant-treated EL4 cells.** *a*, DHA is reduced to AA by GSH, catalyzed by Grx, GST, and PDI and by NADPH-dependent reactions catalyzed by TrxR and 3α-hydroxysteroid dehydrogenase (*3*α-*HSD*). Reduction of GSSG is catalyzed by glutathione reductase (*GR*). *6PGL*, 6-phosphogluconolactone; *6PGLase*, 6-phosphogluconolactonase; *LDH*, lactate dehydrogenase. *b–d*, ratio of lactate singly labeled at C3 to the total labeled lactate concentration. Cells were incubated for 30 min with 11 mm [1,2-^13^C]glucose and 50 μm phenazine methosulfate (*PMS*) (*b*), 1 mm H_2_O_2_ (*c*), or 11 mm DHA (*d*). *e–g*, ratio of ^13^C to ^12^C-labeled 6PG in cells that were incubated for 30 s with 11 mm [U-^13^C]glucose and had been incubated previously for 30 min with 50 μm PMS (*e*), 1 mm H_2_O_2_ (*f*), or 11 mm DHA (*g*). *Error bars*, S.D. (*n* = 3). *, *p* < 0.05; **, *p* < 0.01; ****, *p* < 0.0001.

Cancer cell proliferation promotes the production of reducing equivalents, such as reduced glutathione (GSH) and NADPH, which counteract the effects of relatively high levels of reactive oxygen species (ROS)^5^ (1). The most abundant intracellular antioxidant, GSH, which is typically 0.5–10 mm, can react with hydrogen peroxide in the reaction catalyzed by glutathione peroxidase and can also react directly with ROS in non-enzyme-catalyzed reactions (2). The resulting oxidized glutathione (GSSG) is reduced by NADPH-dependent glutathione reductase, which maintains it in the 5–50 μm range (2). A major source of this NADPH is the cytosolic pentose phosphate pathway (PPP) (3) (Fig. 1*a*). The redox couples GSSG/GSH and NADP^+^/NADPH are therefore a reflection of cell redox state, which increase in the face of increased oxidative stress. Increased ROS production is often associated with chemotherapy-induced apoptosis and is an early event in tumor responses to treatment (4). Depletion of glutathione increases the tumor-specific cytotoxicity of several chemotherapeutic drugs without increasing toxicity to normal tissues (5), and the ability of some cancer cells to maintain a highly reduced intracellular environment has been correlated with tumor aggressiveness and drug resistance (6).

Hyperpolarized ^13^C magnetic resonance spectroscopy (MRS) and spectroscopic imaging (MRSI) have enabled real time measurement of metabolic fluxes *in vivo* by increasing the signal-to-noise ratio by more than 10^4^-fold (7). The most widely used substrate has been [1-^13^C]pyruvate, where the rate of hyperpolarized ^13^C label exchange between the injected labeled pyruvate and endogenous lactate has been shown to be a marker of tumor grade and treatment response (8–11). The technique was translated to the clinic recently with a trial in prostate cancer (12). Lactate can also become labeled following injection of hyperpolarized [U-^2^H,U-^13^C]glucose, and the rate of labeling has been used to assess glycolytic flux in breast cancer cells and yeast *in vitro* (13, 14) and to image glycolytic flux in EL4 murine lymphoma tumors *in vivo* (15). In EL4 tumors, labeling of 6-phosphogluconate (6PG), an intermediate in the PPP, was also observed, suggesting that hyperpolarized [U-^2^H,U-^13^C]glucose might also be used for real time assessment of NADPH production in the PPP and therefore potentially the capability of the tumor cells to resist oxidative stress (15, 16). Another potential approach to assess resistance to oxidative stress is to monitor the rate of reduction of hyperpolarized [1-^13^C]dehydroascorbic acid (DHA) to [1-^13^C]ascorbic acid (AA). DHA reduction can occur spontaneously by reaction with GSH or be catalyzed by the GSH-dependent thiol-disulfide oxidoreductases, glutaredoxin (Grx; EC 1.20.4.1) and protein-disulfide isomerase and by the NADPH-dependent enzymes thioredoxin reductase (TrxR; EC 1.8.1.9) and 3α-hydroxysteroid dehydrogenase (17). Reduction of hyperpolarized [1-^13^C]DHA to [1-^13^C]AA has been detected using ^13^C MRS and MRSI, both *in vitro* and *in vivo*, including in tumors (18–20), where the rate was suggested to depend on the levels of GSH (19–21). Despite the increase in sensitivity to MR detection afforded by hyperpolarization, [1-^13^C]DHA must be used at relatively high concentrations, and therefore DHA is itself an oxidative stressor. DHA has been shown previously, for example, to oxidize GSH, to increase PPP flux (23), and to have adverse effects on the central nervous system (24).

The aim of this study was to determine whether the rate of hyperpolarized [1-^13^C]DHA reduction is dependent only on the levels of GSH or if it also depends on PPP flux. First we showed that DHA treatment of tumor cells *in vitro* produces a similar and rapid increase in PPP flux as the oxidants hydrogen peroxide and phenazine methosulfate (PMS), which is an NADPH-oxidizing agent (25). We then showed that intravenous administration of DHA produces a similarly rapid increase in PPP flux in tumor cells *in vivo*. Changes in PPP flux were assessed using [1,2-^13^C_2_]glucose and measuring label incorporation into lactate in cell and tissue extracts using ^13^C MRS and by measuring label incorporation from [U-^13^C]glucose into 6PG using liquid chromatography tandem mass spectrometry (LC-MS/MS) (26, 27). We also measured 6PG labeling from hyperpolarized [U-^2^H,U-^13^C]glucose in tumors *in vivo* using ^13^C MRS measurements. Next we showed that depletion of the glutathione pool in tumor cells *in vitro* and tumors *in vivo*, using the γ-glutamyl-cysteine synthetase inhibitor buthionine sulfoximine (BSO) (28), led to increased PPP flux and Grx activity and an increased rate of hyperpolarized [1-^13^C]DHA reduction in two different tumor models, thus demonstrating that the rate does not depend only on the levels of GSH. However, DHA led to transient respiratory arrest and cardiac depression in the tumor-bearing animals. Hyperpolarized [1-^13^C]AA, an alternative substrate for assessing oxidative stress that shows no such toxicity, has been observed previously to show no detectable oxidation in tumors (18). We show here that its oxidation is likely to be transport-limited and dependent on intracellular ROS.

## Results

### 

#### 

##### DHA Administration Results in Rapid Increases in the GSSG/GSH Ratio and PPP Flux in Cells and Tumors

First we showed that DHA produced the same rapid increase in PPP flux as the oxidants hydrogen peroxide and PMS (Fig. 1*a*). EL4 murine lymphoma cells were incubated for 30 min with either 50 μm PMS (25), 1 mm hydrogen peroxide (29), or 11 mm DHA (30). Total glutathione levels and the GSSG/GSH ratio were measured by LC-MS/MS (31). Total glutathione was unchanged by PMS and DHA treatment, but there was a small but significant decrease following hydrogen peroxide treatment (Table 1). With all three oxidants, there was a marked increase in the GSSG/GSH ratio, consistent with an increase in oxidative stress (32). Flux into the PPP was assessed by simultaneous incubation with 11 mm [1,2-^13^C_2_]glucose and analysis of the labeling pattern in the resulting lactate using ^13^C NMR (27) (Fig. 1, *b–d*) and by incubation with 11 mm [U-^13^C]glucose 30 min after oxidant treatment and then 30 s later, analyzing label incorporation into 6PG using LC-MS/MS (26) (Fig. 1, *e–g*). Both methods showed a similar and rapid increase in PPP flux with all of the oxidants.

**TABLE 1:** **Glutathione concentration, GSSG/GSH ratio, and GAPDH and G6PDH activity in oxidant-treated EL4 cells and tumors and Colo205 cells** *, *p* < 0.05; **, *p* < 0.01; ***, *p* < 0.001; errors represent S.E.

	50 μm PMS (30 min)		1 mm H_2_O_2_ (30 min)		11 mm DHA (30 min) cells 28 mm DHA (4 min i.v.) tumors	
	−	+	−	+	−	+
**Total glutathione (nmol 10^6^ cells^−1^/mg tumor^−1^)**						
EL4 cells	0.92 ± 0.01 (*n* = 3)	0.82 ± 0.04 (*n* = 3)	0.96 ± 0.02 (*n* = 2)	0.83 ± 0.01** (*n* = 3)	0.56 ± 0.03 (*n* = 3)	0.56 ± 0.04 (*n* = 2)
EL4 tumors					0.72 ± 0.07 (*n* = 4)	0.89 ± 0.06 (*n* = 7)
Colo205 cells	4.57 ± 0.27 (*n* = 3)	4.89 ± 0.19 (*n* = 3)	4.57 ± 0.27 (*n* = 3)	4.62 ± 0.19 (*n* = 3)	4.57 ± 0.27 (*n* = 3)	4.96 ± 0.43 (*n* = 3)

**GSSG/GSH**						
EL4 cells	11.0 ± 0.6 × 10^−3^ (*n* = 3)	24.5 ± 1.1 × 10^−3^*** (*n* = 3)	18.7 ± 0.6 × 10^−3^ (*n* = 2)	40.9 ± 2.7 × 10^−3^** (*n* = 3)	13.8 ± 3.3 × 10^−3^ (*n* = 3)	61.3 ± 0.3 × 10^−3^** (*n* = 2)
EL4 tumors					119 ± 21 × 10^−3^ (*n* = 4)	121 ± 16 × 10^−3^ (*n* = 7)
Colo205 cells	54 ± 11 × 10^−3^ (*n* = 3)	737 ± 56 × 10^−3^*** (*n* = 3)	54 ± 11 × 10^−3^ (*n* = 3)	69 ± 5 × 10^−3^ (*n* = 3)	54 ± 11 × 10^−3^ (*n* = 3)	109 ± 11 × 10^−3^* (*n* = 2)

**GAPDH activity (nmol min^−1^ 10^6^ cells^−1^/mgtumor^−1^)**						
EL4 cells	148 ± 4 (*n* = 3)	149 ± 14 (*n* = 3)	148 ± 4 (*n* = 3)	59 ± 8*** (*n* = 3)	159 ± 10 (*n* = 3)	173 ± 2 (*n* = 3)
EL4 tumors					62 ± 2 (*n* = 4)	51 ± 4 (*n* = 4)
Colo205 cells	299 ± 6 (*n* = 2)	334 ± 50 (*n* = 3)	299 ± 6 (*n* = 2)	286 ± 45 (*n* = 3)	299 ± 6 (*n* = 2)	224 ± 54 (*n* = 3)

**G6PDH activity (nmol min^−1^ mg protein^−1^)**						
EL4 cells	69 ± 8 (*n* = 3)	16 ± 2** (*n* = 3)	69 ± 8 (*n* = 3)	51 ± 12 (*n* = 3)	69 ± 8 (*n* = 3)	39 ± 7* (*n* = 3)
EL4 tumors					61 ± 4 (*n* = 3)	112 ± 4*** (*n* = 4)
Colo205 cells	92 (*n* = 3)	106 ± 5* (*n* = 3)	92.13 ± 1.14 (*n* = 3)	163 ± 9** (*n* = 3)	92 ± 1 (*n* = 3)	81 ± 10 (*n* = 3)

**FIGURE 2.**
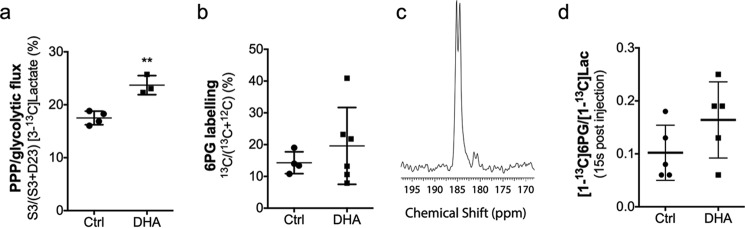
**PPP flux in EL4 tumors treated with DHA.** EL4 tumor-bearing mice were injected with 0.4 ml of 200 mm [1,2-^13^C_2_]glucose (*n* = 4) or 0.4 ml of 200 mm [1,2-^13^C_2_]glucose and 28 mm DHA (*n* = 3). Lactate labeling was measured in tissue extracts by ^13^C NMR. *a*, ratio of lactate singly labeled at C3 to total labeled lactate. *b*, ratio of ^13^C to ^12^C-labeled 6PG in control (*Ctrl*) EL4 tumors (*n* = 4) injected with 0.4 ml of 200 mm [U-^13^C]glucose and EL4 tumors injected simultaneously with 28 mm DHA and 0.4 ml of 200 mm [U-^13^C]glucose (*n* = 6). *c*, ^13^C spectrum of hyperpolarized [1-^13^C]lactate (185.1 ppm) and [1-^13^C]6PG (181.4 ppm) in a control EL4 tumor 15 s after the start of injection of 0.4 ml of 200 mm hyperpolarized [U-^2^H,U-^13^C]glucose. *d*, untreated mice (*Ctrl*, *n* = 5) or mice pretreated 3 min earlier with 28 mm DHA (*n* = 5) before injection of hyperpolarized [U-^2^H,U-^13^C]glucose (200 mm, 0.4 ml). The ratio of the sum of the intensities of the [1-^13^C]6PG resonance to the sum of the intensities of the [1-^13^C]lactate resonance in the first nine spectra is shown. *Error bars*, S.D. **, *p* < 0.01.

Next we assessed the effect of DHA administration on PPP flux *in vivo*. ^13^C MRS measurements of lactate labeling in EL4 tumor extracts from animals injected with DHA and [1,2-^13^C_2_]glucose showed a significant increase in PPP flux as compared with control tumors, although this was less evident than in EL4 cells *in vitro* (Fig. 2*a*). However, the increase in 6PG labeling, measured in tumor extracts in animals injected with [U-^13^C]glucose (Fig. 2*b*) or measured using non-invasive localized ^13^C MRS measurements *in vivo* in animals injected with hyperpolarized [U-^13^C,U-^2^H]glucose (15) (Fig. 2, *c* and *d*), was not significantly different between untreated mice and mice injected with DHA. Unlike EL4 cells, the DHA-treated tumors showed no change in the GSSG/GSH ratio compared with untreated tumors; however, there was a significant increase in the activity of the PPP enzyme, glucose-6-phosphate dehydrogenase (G6PDH) (Table 1), consistent with an increase in oxidative stress (33).

Flux of glutamine carbon via malic enzyme, to lactate, also produces NAPDH for glutathione reduction (34). However, tumor extracts prepared from EL4 tumor-bearing mice injected with [3-^13^C]glutamine showed no significant increase in lactate C2 and C3 labeling following DHA treatment (C2 labeling was 0.18 ± 0.03 μmol min^−1^ g tumor^−1^ in control tumors (*n* = 4) *versus* 0.30 ± 0.09 μmol min^−1^ g tumor^−1^ in DHA-treated tumors (*n* = 4), *p* = 0.26).

These experiments have shown that in using DHA as a probe of the capacity of a cell to resist oxidative stress, it results in an increase in the GSSG/GSH ratio, as it is reduced to AA, and a rapid increase in PPP flux. Next we asked what would happen to the rate of DHA reduction in a tumor cell that had been oxidatively prestressed. We chose to do this by depleting the glutathione pool because this would also allow us to examine how the rate of DHA reduction was related to GSH concentration.

##### Inducing Oxidative Stress in Cells by Glutathione Depletion

**FIGURE 3.**
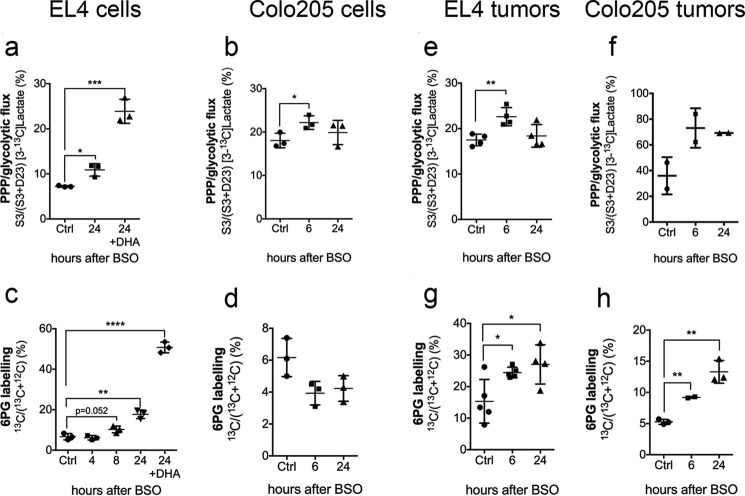
**PPP flux in BSO-treated EL4 and Colo205 cells and tumors.** The ratio of lactate singly labeled at C3 to total labeled lactate concentration in EL4 cells (10^8^) (*a*) and Colo205 cells (10^7^) (*b*) treated with 100 μm BSO and then incubated in RPMI medium containing 11 mm [1,2-^13^C_2_]glucose with or without 11 mm DHA for 30 min. EL4 (*e*) and Colo205 (*f*) tumors were treated with 500 mg kg^−1^ BSO and injected with 0.4 ml of 200 mm [1,2-^13^C_2_]glucose and freeze-clamped 4 min later. Shown is the ratio of ^13^C- to ^12^C-labeled 6PG in extracts of EL4 (*c*) and Colo205 (*d*) cells treated with 100 μm BSO and incubated for 30 s with 11 mm [U-^13^C]glucose with or without 11 mm DHA. Shown are EL4 (*g*) and Colo205 (*h*) tumors treated with 500 mg kg^−1^ BSO and then injected with 0.4 ml of 200 mm [U-^13^C]glucose and freeze-clamped 1 min later. *Error bars*, S.D. *, *p* < 0.05; **, *p* < 0.01; ***, *p* < 0.001; ****, *p* < 0.0001.

BSO sensitizes tumor cells to radiotherapy (35) by depleting glutathione (Fig. 3*a*) (28). Treatment of EL4 and Colo205 cells with BSO decreased glutathione levels, although the levels of glutathione were much higher in Colo205 cells (Table 2). In both cell lines, there was a marked decrease in the GSSG/GSH ratio (Table 2), indicating up-regulation of pathways responsible for maintaining glutathione in a reduced state. This can be explained by an increase in PPP flux. At 24 h following BSO treatment, PPP flux in EL4 cells, assessed from measurements of lactate labeling, was increased by 1.5-fold (Fig. 3*a*), which was increased a further ∼2.2-fold by the addition of DHA (Fig. 3*b*). ^13^C label incorporation into 6PG was increased by more than 2-fold at 24 h after BSO treatment (*n* = 3, *p* = 0.0019), which was increased a further 4-fold by the addition of DHA (*n* = 3, *p* < 0.0001) (Fig. 3*d*). GAPDH and G6PDH activities were unchanged; however, Grx activity increased significantly at 24 h after BSO treatment (Table 2). In Colo205 cells, BSO treatment for 6 h led to a significant increase in PPP flux, as assessed from measurements of lactate labeling (Fig. 3*c*), but there was no significant change in 6PG labeling (Fig. 3*e*). Both GAPDH and G6PDH activities increased significantly at 24 h following BSO treatment (Table 2). In summary, these experiments have shown that oxidatively prestressing cells by glutathione depletion results in up-regulation of the PPP and a lower steady state GSSG/GSH ratio. DHA administration, as was shown in non BSO-treated cells, resulted in a rapid increase in PPP flux.

**TABLE 2:** **Glutathione concentration, GSSG/GSH ratio, and GAPDH, G6PDH, and GRX enzyme activities in BSO-treated EL4 and Colo205 cells and tumors** *, *p* < 0.05; **, *p* < 0.01; ****, *p* < 0.0001; errors represent S.E. +DHA, mice injected with 28 mm DHA i.v. 5 min before tissue collection. Ctrl, control.

	EL4 cells (100 μm BSO)	EL4 tumors (500 mg kg^−1^ BSO (i.p.))	Colo205 cells (100 μm BSO)	Colo205 tumors (500 mg kg^−1^ BSO (i.p.))
**Total glutathione (nmol 10^6^ cells^−1^/mg tumor^−1^)**	Ctrl: 0.62 ± 0.01 (*n* = 3)	Ctrl: 0.96 ± 0.04 (*n* = 5)	Ctrl: 4.57 ± 0.27 (*n* = 3)	Ctrl: 2.23 ± 0.31 (*n* = 4)
	4 h: 0.16 ± 0.01**** (*n* = 3)	6 h: 0.56 ± 0.08** (*n* = 5)	6 h: 1.77 ± 0.10*** (*n* = 3)	6 h: 1.80 ± 0.29 (*n* = 4)
	8 h: 0.16 ± 0.01**** (*n* = 3)	24 h: 1.11 ± 0.06 (*n* = 4)	24 h: 1.29 ± 0.04** (*n* = 2)	24 h: 1.83 ± 0.31 (*n* = 3)
	24 h: 0.15 ± 0.01**** (*n* = 3)	24 h + DHA: 0.93 ± 0.26 (*n* = 3)		
**GSSG/GSH**	Ctrl: 68 ± 9 × 10^−3^	Ctrl: 57 ± 9 × 10^−3^ (*n* = 5)	Ctrl: 54 ± 11 × 10^−3^ (*n* = 3)	Ctrl: 104 ± 30 × 10^−3^ (*n* = 4)
	4 h: 0.0*^a^*	6 h: 29 ± 4 × 10^−3^* (*n* = 5)	6 h: 10 ± 2 × 10^−3^* (*n* = 3)	6 h: 123 ± 35 × 10^−3^ (*n* = 4)
	8 h: 0.0*^a^*	24 h: 33 ± 5 × 10^−3^ (*n* = 4)	24 h: 0.2 ± 0.2 × 10^−3^** (*n* = 2)	24 h: 82 ± 11 × 10^−3^ (*n* = 3)
	24 h: 0.0*^a^*	24 h + DHA: 90 ± 10 × 10^−3^* (*n* = 3)		
**GAPDH activity (nmol min^−1^ 10^6^ cells^−1^/mg tumor^−1^)**	Ctrl: 892 ± 105 (*n* = 3)	Ctrl: 69 ± 21 (*n* = 4)	Ctrl: 299 ± 6 (*n* = 2)	Ctrl: 339 ± 31 (*n* = 3)
	4 h: 709 ± 51 (*n* = 3)	6 h: 39 ± 16 (*n* = 4)	6 h: 308 ± 21 (*n* = 3)	6 h: 271 ± 73 (*n* = 3)
	8 h: 837 ± 37 (*n* = 3)	24 h: 65 ± 9 (*n* = 4)	24 h: 346 ± 5** (*n* = 4)	24 h: 265 ± 49 (*n* = 4)
	24 h: 734 ± 85 (*n* = 3)			
**G6PDH activity (nmol min^−1^mg protein^−1^)**	Ctrl: 69 ± 8 (*n* = 3)	Ctrl: 61 ± 34 (*n* = 3)	Ctrl: 92 ± 1 (*n* = 3)	Ctrl: 44 ± 2 (*n* = 3)
	4 h: 59 ± 3 (*n* = 3)	6 h: 82 ± 8 (*n* = 3)	6 h: 105 ± 5 (*n* = 3)	6 h: 36 ± 4 (*n* = 3)
	8 h: 59 ± 7 (*n* = 3)	24 h: 85 ± 4** (*n* = 3)	24 h: 127 ± 11* (*n* = 3)	24 h: 48 ± 1 (*n* = 4)
	24 h: 73 ± 4 (*n* = 2)			
**GRX activity (nmol min^−1^ mg protein^−1^)**	Ctrl: 76 ± 6 (*n* = 3)	Ctrl: 12 ± 2 (*n* = 9)	Ctrl: 79 ± 7 (*n* = 3)	Ctrl: 68 ± 6 (*n* = 4)
	4 h: 84 ± 13 (*n* = 3)	24 h: 17 ± 2* (*n* = 7)	6 h: 137 ± 28 (*n* = 3)	6 h: 72 ± 12 (*n* = 3)
	8 h: 101 ± 9 (*n* = 3)	24 h + DHA: 21 ± 2* (*n* = 3)	24 h: 140 ± 40 (*n* = 3)	24 h: 77 ± 7 (*n* = 3)
	24 h: 105 ± 4* (*n* = 3)			

*^a^* GSSG was below the quantification limit.

Next we investigated the effects of glutathione depletion in EL4 and Colo205 tumors. BSO is cleared within 24 h of infusion in mice and causes no obvious toxicity (36). In EL4 tumors 6 h after BSO treatment, there was a significant decrease in glutathione content (*n* = 5, *p* = 0.0013) and a 2-fold decrease in the GSSG/GSH ratio (Table 2). By 24 h, glutathione levels had recovered, whereas the GSSG/GSH ratio remained lower than in controls, although this was not significant (Table 2). In animals treated 24 h previously with BSO and injected with hyperpolarized [1-^13^C]DHA immediately before freeze clamping of the tumor, glutathione levels were similar to those in control tumors; however, the GSSG/GSH ratio was significantly higher (*n* = 3, *p* = 0.048), reflecting rapid reduction of the DHA by GSH (Table 2). Measurements of lactate labeling in tumor extracts showed that 6 h after BSO treatment, there was a significant increase in PPP flux compared with untreated tumors (*n* = 4, *p* = 0.0051) (Fig. 3*e*), which was confirmed by increased ^13^C labeling of 6PG (Fig. 3*g*) at 6 and 24 h after BSO treatment. As in the experiments on the cells *in vitro*, this increased PPP flux can explain the lower GSSG/GSH ratio. Glycolytic flux decreased from 1.19 ± 0.09 to 0.55 ± 0.19 μmol min^−1^ g^−1^ (*n* = 4, *p* = 0.0009) at 6 h but increased to 0.93 ± 0.10 μmol min^−1^ g^−1^ (*n* = 4, *p* = 0.04) at 24 h after BSO treatment. At 24 h, there was increased fractional labeling of the glycolytic intermediates 3-phosphoglycerate and phosphoenolpyruvate (3PG, 11.3 ± 2.9% in control *versus* 29.9 ± 4.6% in treated tumors, *n* = 4, *p* = 0.0136; PEP, 51.4 ± 5.4% in control *versus* 76.2 ± 4.3% in treated tumors, *n* = 4, *p* = 0.0116). GAPDH activity was unchanged, but there was an increase in G6PDH activity (Table 2), again indicative of oxidative stress (33) and consistent with data indicating increased PPP flux (Fig. 3, *e* and *g*). Of the enzymes involved in DHA reduction, Grx activity increased 24 h after BSO treatment (Table 2), as was observed in the cells, both with (*n* = 3, *p* = 0.02) and without injection of DHA (*n* = 7, *p* = 0.045). There were no changes in the activities of TrxR (69.4 ± 1.0 nmol min^−1^ mg tumor^−1^ in control (*n* = 6) *versus* 64.0 ± 1.3 nmol min^−1^ mg tumor^−1^ in BSO-treated tumors (*n* = 5)) and GST (40 ± 1 nmol min^−1^ mg tumor^−1^ in control (*n* = 6) *versus* 39 ± 1 nmol min^−1^ mg tumor^−1^ in BSO-treated tumors (*n* = 5)).

BSO treatment of Colo205 tumors had no significant effect on glutathione levels or GSSG/GSH ratio (Table 2). There were, however, significant increases in 6PG labeling at 6 and 24 h after BSO treatment, indicating an increase in PPP flux, although this was not reflected in lactate labeling (Fig. 3, *f* and *h*). There were no significant changes in the activities of GAPDH, G6PDH, or Grx (Table 2).

##### Detecting Oxidative Stress in BSO-treated EL4 and Colo205 Tumors Using Hyperpolarized [1-^13^C]Dehydroascorbic Acid

**FIGURE 4.**
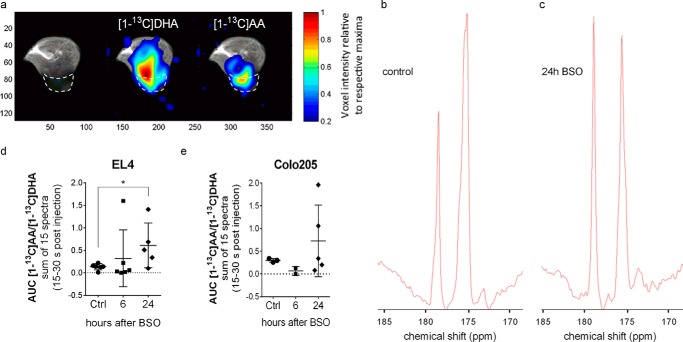
**DHA reduction rate in EL4 and Colo205 tumors.** *a*, chemical shift images of [1-^13^C]DHA and [1-^13^C]AA in an EL4 tumor-bearing mouse 27 s after injecting 0.2 ml of 28 mm hyperpolarized [1-^13^C]DHA. Color intensities were normalized to the respective maxima in the images. *b–e*, ^13^C spectroscopic measurements of DHA reduction in EL4 and Colo205 tumors treated with 500 mg kg^−1^ BSO and then injected with 0.4 ml of 28 mm hyperpolarized [1-^13^C]DHA 6 or 24 h later. Spectra were acquired 15 s after the start of injection. Representative spectra, which are the sum of the 15 spectra acquired during the first 15 s, are shown for a control EL4 tumor (*b*) and an EL4 tumor 24 h after BSO treatment (*c*). The signal at 175.3 ppm is from [1-^13^C]DHA, and the signal at 179 ppm is from [1-^13^C]AA. Shown is the ratio of the sum of the hyperpolarized [1-^13^C]DHA and [1-^13^C]AA signals in the first 15 s of data acquisition (15 spectra) for EL4 tumors (*d*) and Colo205 tumors (*e*). *Error bars*, S.D. *, *p* < 0.05.

Non-invasive ^13^C MR spectroscopic measurements with hyperpolarized [1-^13^C]DHA were performed in the same cohort of animals as used for GSSG/GSH and enzyme activity measurements. A separate cohort of animals was used for measurements of PPP flux. Hyperpolarized [1-^13^C]DHA has been shown previously to be rapidly reduced in tumors *in vivo*(18–20). Consistent with these previous observations, ^13^C spectroscopic images showed a more general distribution of [1-^13^C]DHA throughout the animal, whereas [1-^13^C]AA, produced by intracellular reduction of DHA, was localized mainly to the tumor region (Fig. 4*a*). Oxidatively prestressing EL4 tumors by BSO treatment increased the rate of hyperpolarized [1-^13^C]DHA reduction, which, although highly variable, was ∼3.8-fold higher in tumors treated 24 h previously with BSO than in control tumors (*p* = 0.044) (Fig. 4, *b–d*). At this time point, there was no difference in total glutathione content and GSH concentration (Table 2) between control and BSO-treated tumors, but there was a significant increase in PPP flux (Fig. 3*g*). ^13^C NMR spectra of extracts prepared from these tumors, where the tumors were freeze-clamped at ∼5 min after injection of the hyperpolarized [1-^13^C]DHA, showed that there was 70.5 ± 40.5 nmol g^−1^ [1-^13^C]DHA in 24-h BSO-treated tumors (*n* = 2) and 97.5 ± 34.5 nmol g^−1^ in control tumors (*n* = 2). [1-^13^C]AA was undetectable, although this may reflect oxidation of AA during perchloric acid extraction. Assuming that this is the concentration of [1-^13^C]DHA present at the time the ^13^C MRS measurements were made *in vivo* and using the [1-^13^C]AA/[1-^13^C]DHA ratio at 30 s after injection of hyperpolarized [1-^13^C]DHA, this equates to a [1-^13^C]DHA reduction rate of 0.42 ± 0.15 and 1.43 ± 0.82 nmol g^−1^ s^−1^ in control tumors and 24-h BSO-treated tumors, respectively. In Colo205 tumors, the hyperpolarized [1-^13^C]DHA reduction rate at 24 h after BSO treatment was also highly variable, but in this case the increase was not significant (Fig. 4*c*). Although in these BSO-treated Colo205 tumors there was no measurable decrease in the GSSG/GSH ratio, there was nevertheless a significant increase in PPP flux (Fig. 3, *f* and *h*).

**FIGURE 5.**
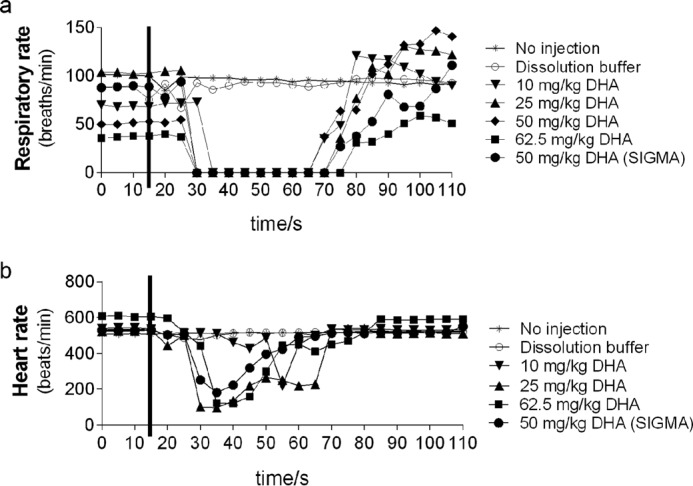
Respiratory rate (*a*) and heart rate (*b*) of C57BL/6 mice (no injection, *) or injected with dissolution buffer (○) or 10 (▾), 25 (▴), 50 (♦), or 62.5 mg kg^−1^ (■) DHA, produced by charcoal oxidation of ascorbic acid or 50 mg kg^−1^ DHA from Sigma-Aldrich (●). Each condition was tested in a separate mouse. The *black lines* indicate time of injection.

Hyperpolarized [1-^13^C]DHA led to transient respiratory arrest and cardiac depression in these mice, which was dose-dependent (Fig. 5). Respiratory and cardiovascular effects of DHA have been observed previously following intravenous injection in rats (24).

##### Detecting Oxidative Stress with Hyperpolarized [1-^13^C]Ascorbic Acid

**FIGURE 6.**
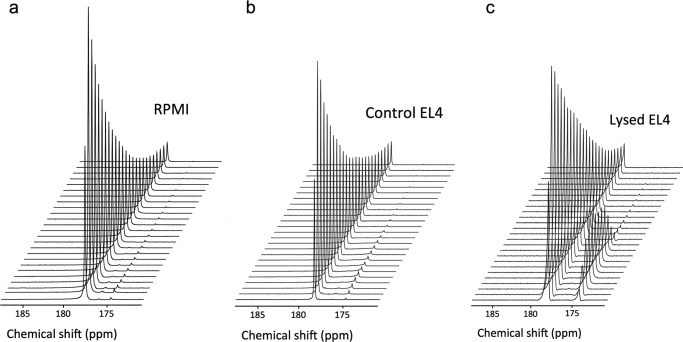
**Oxidation of hyperpolarized [1-^13^C]AA in EL4 tumor cell suspensions.** Shown is a representative time course of ^13^C signals from hyperpolarized [1-^13^C]AA (179.0 ppm) and [1-^13^C]DHA (175.3 ppm) in RPMI medium (*a*) and in a suspension of 10^8^ (2.5 × 10^7^ ml^−1^) intact (*b*) or lysed (*c*) EL4 cells.

Because DHA has adverse, although transient, effects on mouse physiology, we also explored whether oxidative stress could be assessed using [1-^13^C]AA, which is nontoxic and used clinically in intravenous infusions (37). We had shown previously that hyperpolarized [1-^13^C]AA can be injected into mice and detected in EL4 tumors (18). However, although oxidation of hyperpolarized [1-^13^C]AA to [1-^13^C]DHA was observed *in vitro*, no [1-^13^C]DHA was observed in EL4 tumors *in vivo*. One explanation for this is that any DHA produced is rapidly re-reduced to AA (18). To investigate this further, we examined the factors affecting AA oxidation. Hyperpolarized [1-^13^C]AA reacted only slowly with hydrogen peroxide. Fitting the hyperpolarized [1-^13^C]DHA peak intensity gave a pseudo-first order rate constant for the oxidation of AA by 100 μm H_2_O_2_ of 9 × 10^−3^ s^−1^ and, therefore, a second order rate constant of 90 m^−1^ s^−1^, which is similar to values measured previously (38) but is orders of magnitude slower than the second order rate constant for the reaction of AA with superoxide (39). When [1-^13^C]AA was added to an EL4 tumor cell suspension, a low rate of oxidation was observed (Fig. 6*b*), as reported previously (18). The oxidation rate in the cell suspension was similar to that observed in cell culture medium (RPMI) alone (Fig. 6*a*). However, when the cells were lysed, the rate of AA oxidation was much higher (Fig. 6*c*), 2.18 mmol liter^−1^ s^−1^ *versus* 4.96 mmol liter^−1^ s^−1^.

## Discussion

There has been considerable interest in the development of imaging methods that could be used to image oxidative stress and cellular redox state non-invasively *in vivo* (40). Glutathione has been measured using ^1^H MRS (41); T_1_-weighted MRI and EPR imaging of injected nitroxides have been used, in preclinical studies, to assess tissue redox status (42–44); and an EPR method that measures extracellular pH, redox status, and intracellular GSH concentration has been described (45). MRI probes of ROS based on T_1_-shortening or chemical exchange saturation transfer and redox-active PET tracers have also been developed (46, 47); however, most agents are not taken up by cells and lack sensitivity for biologically relevant redox ranges. We have described here a dynamic ^13^C magnetic resonance spectroscopy method for imaging the capacity of tumors *in vivo* to resist oxidative stress using hyperpolarized [1-^13^C]DHA.

We have shown that DHA acts as a cellular oxidant in cells *in vitro*, producing increases in the GSSG/GSH ratio similar to those produced by treatment of the cells with the oxidants PMS and hydrogen peroxide (Table 1). The increase in the GSSG/GSH ratio was accompanied by a marked and rapid increase in PPP flux, assessed using either LC-MS/MS measurements of 6PG labeling in cells incubated with [U-^13^C]glucose or ^13^C NMR measurements of lactate labeling in cells incubated with [1,2-^13^C_2_]glucose (Fig. 1). DHA has been shown previously to oxidize GSH and increase PPP flux in primary rat cortical neurons (23). The increased PPP flux in cells treated with hydrogen peroxide can be explained by a reduction in GAPDH activity (29), whereas in cells treated with PMS or DHA, the increase in flux is, in part, consistent with an increase in G6PDH activity (33) (Table 1). DHA treatment of EL4 tumors also resulted in a small but significant increase in PPP flux, as determined from ^13^C NMR measurements of lactate labeling in tumor extracts. However, the increase in 6PG labeling, measured in tumor extracts using LC-MS/MS measurements and measured non-invasively *in vivo* using ^13^C MRS in animals injected with hyperpolarized [U-^2^H,U-^13^C]glucose, was not significant (Fig. 2). The much smaller increase in the measured PPP flux in EL4 tumors, as compared with the cells, reflects a lower level of oxidative stress induced by DHA administration. This was evident from the GSSG/GSH ratio, which was increased ∼4.4-fold in the cells following DHA administration, whereas there was no significant change in the tumors (Table 1), although the ratio in the tumors was already ∼10-fold higher than in the cells. Nevertheless, the tumors were evidently oxidatively stressed by DHA administration, because they showed a nearly 2-fold increase in G6PDH activity (Table 1), consistent with the small but significant increase in PPP flux. Radiotherapy, which generates increased levels of ROS (48), increases G6PDH activity in cancer cells within 10 min of exposure, and this has been attributed to ROS-dependent ATM kinase activation and subsequent phosphorylation of Hsp27, which binds to G6PDH directly and enhances its activity (33). Pathways other than the PPP can contribute to intracellular NADPH production and thus to reduction of GSSG and DHA, including flux through malic enzyme (34). However, there was no evidence in this study that NADPH came from this pathway.

Next, we determined whether the rate of DHA reduction to AA could be used to assess the increased oxidative stress induced by depleting the glutathione pool using BSO treatment. Treatment of EL4 and Colo205 cells decreased glutathione content and markedly reduced the GSSG/GSH ratio (Table 2), implying that the cells had responded to the oxidative stress imposed by glutathione depletion by up-regulating those pathways responsible for NADPH production, which maintained the glutathione pool in a more reduced state. Consistent with this were measurements of increased PPP flux in EL4 cells, assessed from measurements of both lactate and 6PG labeling. The addition of DHA to these cells, which imposed an acute oxidative load on the cells, resulted in a further marked increase in PPP flux (Fig. 3, *a* and *c*). Colo205 cells showed less evidence for increased PPP flux following BSO treatment, with only a small but nevertheless significant increase at 6 h, determined from changes in lactate labeling (Fig. 3*b*). The more modest increase in PPP flux in Colo205 cells may reflect the much higher glutathione concentration in these cells (Table 2), making them less dependent on the PPP for buffering the GSH concentration (see Fig. 1).

Treatment of EL4 tumor-bearing mice with BSO decreased glutathione by 6 h after treatment and decreased the GSSG/GSH ratio by 2-fold. This shows, similarly to EL4 cells *in vitro*, that the cells had responded to the oxidative stress imposed by BSO treatment by increasing PPP flux, the increase in NADPH production leading to a lower steady state GSSG/GSH ratio. Glutathione depletion also increased Grx activity at 24 h after BSO treatment. BSO has been shown previously to decrease glutathione levels by 40% in RIF-1 tumors 6 h after injection (43) and to increase Grx activity, leading to increased GSSG reduction and a decreased GSSG/GSH ratio (43). That the tumors were oxidatively stressed is further indicated by the increase in G6PDH activity (33) and the increased labeling of 3PG and PEP, which is indicative of oxidation and consequent inhibition of PKM2 (49). The inferred decrease in PKM2 activity was consistent with the measured decrease in glycolytic flux. Although BSO treatment of Colo205 cells had effects on the levels of glutathione similar to those observed in EL4 cells, in Colo205 tumors, there was no significant depletion of glutathione, change in the GSSG/GSH ratio, or change in enzyme activities. Nevertheless, there was an increase in PPP flux evident from both lactate (Fig. 3*g*) and 6PG (Fig. 3*i*) labeling, which was significant in the latter case.

Injection of hyperpolarized [1-^13^C]DHA into EL4 tumor-bearing mice resulted in its reduction to [1-^13^C]AA, as was observed previously (18), and this reduction was concentrated in the tumor region (Fig. 4*a*). Although the data were highly variable, oxidatively prestressing EL4 tumors by BSO treatment resulted in a ∼3.8-fold increase in the rate of DHA reduction at 24 h after BSO treatment. Previous studies have suggested that the rate of DHA reduction is dependent on the levels of GSH (19–21). These data show that in this tumor model and under these conditions, this is not the case because at 24 h after BSO treatment there was no significant change in the steady state GSH concentration (Table 2). Instead, there was an increased rate of NADPH production, resulting from increased PPP flux, and an increase in Grx activity. This increase in the rate of NADPH production will maintain the GSH level by increasing the rate of GSSG reduction and increasing the rate of DHA reduction catalyzed by GSH-dependent Grx. The estimated rates of DHA reduction in control tumors (0.42 ± 0.15 nmol g^−1^ s^−1^) and tumors 24 h after BSO treatment (1.43 ± 0.82 nmol g^−1^ s^−1^) are comparable with the rate of GSH oxidation estimated previously in erythrocytes (0.28 μm s^−1^) (50). Although a considerable assumption was made in estimating the rate of DHA reduction, this is consistent with GSH being an important reducing agent for DHA (see Fig. 1). At 6 h after BSO treatment, there was no increase in the rate of DHA reduction, although PPP flux was still elevated. However, at this time point, the GSH concentration was decreased significantly, suggesting that the effect of increased NADPH production on the rate of DHA reduction might have been offset at 6 h by the decrease in GSH concentration. BSO-stressed Colo205 tumors, which also showed a significant increase in PPP flux, showed a similar pattern to EL4 tumors in the rate of DHA reduction at 6 and 24 h and decrease in GSH concentration at 6 h after BSO-treatment, although in this case the changes were not significant. The relative roles of GSH- and NADPH-dependent DHA reductases in reducing DHA is debated and has been shown previously to vary between different cell types *in vitro* (51, 52). We have shown here, in tumor cells *in vivo*, that this may also vary within individual tumor cell types under different metabolic conditions.

While providing a potentially powerful real time measurement of the reductive potential of tumors in preclinical studies, the transient respiratory arrest induced by DHA (Fig. 5) represents a challenge to its translation to the clinic. Intravenous injection of DHA in rats was shown previously to lead to hyperactivity and a mixed parasympathetic/sympathetic effect on the nervous system (24). Animals in this previous study died of respiratory failure at an LD_50_ of 320 mg kg^−1^, whereas we observed a transient respiratory arrest in mice at a dose of only 10 mg kg^−1^, suggesting that there may be species-specific differences in its effects. This toxicity might be overcome by preinjecting animals with similar doses of DHA as used for imaging, because this has been shown to improve tolerance to DHA (24) and has been used in previous preclinical studies of hyperpolarized [1-^13^C]DHA (19). However, because DHA is itself a strong oxidant and has been shown here to lead to changes in the GSSG/GSH ratio and to increased PPP flux, these preinjections may have the same effect as BSO and increase the rate of reduction of hyperpolarized [1-^13^C]DHA injected subsequently.

An alternative and less toxic way to assess oxidative stress in tissues, which has clinical potential, would be to observe the rate of AA oxidation; AA is already infused into patients, achieving serum concentrations of 50 mm ascorbic acid (37). However, hyperpolarized [1-^13^C]AA is oxidized only slowly by intact EL4 cells (Fig. 6), and we showed previously that there was no detectable oxidation of AA in EL4 tumors *in vivo* (18). This can be explained by the slow reaction of AA with extracellular H_2_O_2_ and the fact that many cell types cannot take up AA (53). Lysis of EL4 cells resulted in an increased rate of AA oxidation (Fig. 6), which can be explained by increased access of AA to intracellular superoxide (O_2_^˙̄^), which is produced mainly by the mitochondria and NADPH oxidases and is found mostly intracellularly (54). The addition of H_2_O_2_ to U937 cells was shown not to oxidize AA, whereas the addition of an O_2_^˙̄^-generating system resulted in its oxidation (55).

In conclusion, we have shown that the rate of reduction of hyperpolarized [1-^13^C]DHA is sensitive to changes in a tumor's capacity to resist oxidative stress and that this is related not only to the levels of glutathione, as suggested previously (19, 20, 21), but also to changes in PPP flux and Grx activity. The PPP provides for dynamic buffering of the GSH pool, where the addition of DHA results in an immediate increase in the GSSG/GSH ratio (Table 2) and an increase in PPP flux (Fig. 3, *a* and *c*). However, the transient toxicity of DHA will limit if not prevent its potential translation into the clinic.

## Experimental Procedures

### Materials

All materials were purchased from Sigma-Aldrich UK unless stated otherwise.

### Cell Culture and Tumor Induction

EL4 murine lymphoma cells and Colo205 human colorectal cancer cells (ATCC, Manassas, VA) were grown in RPMI 1640 medium (Gibco, Paisley, UK) supplemented with 10% heat-inactivated FBS (Gibco) and 2 mm l-glutamine. Colo205 cells were genotyped by short tandem repeat (STR) genetic profiling using the Power Plex_16HS_Cell Line panel and analyzed using Applied Biosystems Gene Mapper ID version 3.2.1 software (Genetica DNA Laboratories, LabCorp Specialty Testing Group) and overlapped 100% with the Colo205 cell line published on the DSMZ STR database. EL4 cells were STR-genotyped by DDC Medical. EL4 tumors were induced by implanting 5 × 10^6^ cells subcutaneously into the flanks of 6–8-week-old female C57BL/6 mice (Charles River, Ltd., Wilmington, MA) and grown for ∼10 days, when they reached ∼1 cm^3^. Colo205 tumors were induced by implanting 10^7^ cells in 6–8-week-old female BALB/c nude mice (Charles River) and grown for ∼15 days, when they reached ∼1 cm^3^. Experiments complied with licenses issued under the Animals (Scientific Procedures) Act of 1986. Protocols were approved by the Cancer Research UK Cambridge Institute Animal Welfare and Ethical Review Body.

### Measurements of PPP Flux

PPP flux was assessed either using [1,2-^13^C_2_]glucose and measuring label incorporation into lactate using ^13^C NMR (27) or using [U-^13^C]glucose and measuring label incorporation into 6PG using LC-MS/MS (26).

#### 

##### Measurements using [1,2-^13^C_2_]Glucose

EL4 cells (10^8^) and Colo205 cells (10^7^) were incubated with 11 mm [1,2-^13^C_2_]glucose for 30 min at 37 °C. Medium was collected by centrifugation and snap-frozen in liquid nitrogen. Tumor-bearing mice were injected intravenously with 0.4 ml of 200 mm [1,2-^13^C_2_]glucose or 0.4 ml of 200 mm [1,2-^13^C_2_]glucose and 28 mm DHA, and the animals were sacrificed and tumors were excised rapidly 4 min later. The tumors were then freeze-clamped immediately with liquid nitrogen-cooled tongs, metabolites were extracted with 7% perchloric acid, and the extracts were neutralized subsequently with KOH. Cell medium and tumor extracts were freeze-dried, and the lyophilized samples were dissolved in 20 mm phosphate buffer containing 10% ^2^H_2_O and 10 mm ^13^C urea for ^13^C NMR analysis.

##### Measurements Using [U-^13^C]Glucose

EL4 and Colo205 cells (10^7^) were incubated with 11 mm [U-^13^C]glucose for 30 s and then quenched in ice-cold methanol. Tumor-bearing mice were injected intravenously with 0.4 ml of 200 mm [U-^13^C]glucose with or without 28 mm DHA; the animals were then sacrificed, and tumors were excised rapidly at 1 min after injection and then freeze-clamped immediately. Cells were extracted at 5 × 10^7^ ml^−1^, and tumors were excised at 50 mg ml^−1^ in ice-cold 75:25 methanol/acetonitrile containing 0.2% formic acid using metal bead-containing tubes on a Precellys24 homogenizer coupled to a Cryolys® cooler (Stretton Scientific, Stretton, UK) at 4 °C. A second extraction was performed with 200 μl of water, and the organic and aqueous extracts were mixed. Solvent was removed by evaporation, and the extracts were dissolved in 0.75% octylamine in HPLC grade water. LC-MS/MS measurements of ^13^C-labeling of 6PG, 3PG, and PEP were based on a method published previously (26). Analytes were separated using octylamine/acetonitrile gradients on an ACQUITY UPLC^TM^ BEH130 C18 ID column (Waters, Elstree, UK) at 30 °C and detected using a triple quadrupole TSQ Vantage mass spectrometer with an Accela UHPLC system (Thermo Scientific, Loughborough, UK) fitted with a HESI probe with a source temperature of 320 °C.

### Measurements of Reduced and Oxidized Glutathione

GSH and GSSG were measured as described (31). Cells were extracted at 5 × 10^7^ cells ml^−1^, and tumors were homogenized at 50 mg ml^−1^ with 25:75 water/methanol containing 0.025 mm sodium borate, 0.25 mm EDTA, and 1.25 mm 4-fluoro-7-sulfamoylbenzofurazan to derivatize GSH. GSH and GSSG were separated on an Acquity UPLC® HSS T3 column (Waters), and their ions were identified from their specific mass transition in multiple reaction-monitoring mode and from their retention time, using a triple quadrupole TSQ Vantage mass spectrometer with Accela UHPLC system (Thermo Scientific) fitted with a HESI probe with a source temperature of 320 °C. Glutathione-glycine-^13^C_2_,^15^N was added as an internal standard.

### Enzyme Activity Assays

GAPDH (EC 1.2.1.13) activity was measured using the KDalert® kit (Thermo Fisher Scientific, Hemel Hempstead, UK) at 22 °C. Cells were resuspended in lysis buffer at 10^7^ cells ml^−1^, and tumors were freeze-clamped and extracted at 50 mg ml^−1^. G6PDH (EC 1.1.1.49) activity was assayed at 22 °C, as described (56). Tumors were extracted at 200 mg ml^−1^, and cells were extracted at 10^7^ cells ml^−1^ in 50 mm HEPES buffer containing 100 mm KCl, 10 mm phosphate buffer, 10 mm MgCl_2_^−^, 1 mm dithiothreitol, and protease inhibitor mixture (Roche Applied Science) in a Precellys24 homogenizer coupled to a Cryolys® cooler (Stretton Scientific) at 4 °C. Protein content was measured using Direct Detect® (Millipore, Billerica, MA). Grx activity was measured using a 2-hydroxyethyl disulfide coupled assay (57). TrxR activity was measured in a linked reaction with 5,5′-dithiobis-(2-nitrobenzoic acid) and GST (EC 2.5.1.18) activity with 2,4-dinitrochlorobenzene using assay kits (Sigma-Aldrich). For Grx, TrxR, and GST assays, tumor and cell samples were extracted with 50 mm potassium phosphate buffer, pH 7, containing 0.5 mm EDTA and protease inhibitor mixture (Roche Applied Science) in a Precellys24 homogenizer coupled to a Cryolys® cooler (Stretton Scientific) at 4 °C. Protein content was measured using Bradford reagent (Bio-Rad, Hemel Hempstead, UK).

### Measurements of Malic Enzyme Flux

EL4 tumor-bearing mice were injected intravenously with 0.4 ml of 100 mm [3-^13^C]glutamine with or without 28 mm DHA. After 4 min, tumors were rapidly excised, freeze-clamped, extracted with 7% perchloric acid, and neutralized with KOH. Freeze-dried extracts were dissolved in 600 μl of 20 mm phosphate buffer, pH 7, with 10% ^2^H_2_O and 10 mm ^13^C urea. ^13^C NMR spectra were acquired on a 600-MHz spectrometer (Bruker BioSpin, Rheinstetten, Germany) at 300 K with a TR of 3 s and 12,000 scans.

### Hyperpolarization of [U-^2^H,U-^13^C]Glucose, [1-^13^C]Ascorbic Acid, and [1-^13^C]Dehydroascorbic Acid

A 5 m solution of [U-^2^H,U-^13^C]glucose containing 27 mm OX063 (GE Healthcare) and 1.3 mm gadolinium chelate (Dotarem, Guerbet, France) was hyperpolarized using a Hypersense polarizer (Oxford Instruments Molecular Biotools Ltd., Abingdon, UK) at ∼1.3 K (16). Dissolution was performed after ∼90 min using 4 ml of 50 mm NaCl in ^2^H_2_O containing 1 mm EDTA, yielding a final concentration of 200 mm [U-^2^H,U-^13^C]glucose. [1-^13^C]AA (Omicron Biochemicals, Inc., South Bend, IN) (10 mg) was dissolved in 50 μl of DMSO-*d*_6_ (Cambridge Isotope Laboratories, Tewksbury, MA) with 14.8 mm trityl radical (OX063; GE Healthcare) and 1.4 mm gadolinium chelate (Dotarem). The sample was beaded in liquid nitrogen and polarized (18). After 120 min, dissolution was performed using 3.2 ml of H_2_O, and the was solution neutralized with 0.8 ml of 200 mm phosphate buffer containing 400 mm NaCl and 1.8 mm EDTA, giving a final concentration of 14 mm [1-^13^C]AA. [1-^13^C]AA was oxidized to [1-^13^C]DHA, as described (18). A 1 m solution of [1-^13^C]DHA in DMSO-*d*_6_ (Cambridge Isotope Laboratories) containing 27 mm OX063 (GE Healthcare) and 1.4 mm gadolinium chelate (Dotarem) was hyperpolarized as described (18). For the imaging experiments, the DHA was dissolved in dimethyl-^13^C_2_, sulfoxide-*d*_6_ (Isotec, Miamisburg, OH) (22). Dissolution was performed after ∼90 min using 5 ml of H_2_O, and the solution was neutralized with 1 ml of 200 mm phosphate buffer containing 400 mm NaCl and 1.8 mm EDTA, giving a final [1-^13^C]DHA concentration of 28 mm.

### ^13^C Magnetic Resonance Spectroscopy and Spectroscopic Imaging in Vivo

Mice were anesthetized with 2% isoflurane and placed inside a 7 T spectrometer (Agilent, UK), and their body temperature was maintained at 37 °C. Animals were injected via a tail vein cannula with 0.4 ml of the dissolution fluid, and data acquisition started 15 s after the start of injection. Slice-selective ^13^C spectra (8-mm slice) from the tumor were acquired using an actively decoupled dual-tuned ^13^C/^1^H volume transmit coil and a 20-mm ^13^C receive surface coil (Rapid Biomedical, Rimpar, Germany). For experiments with glucose, spectra were acquired with a nominal flip angle of 22°, TR 100 ms, and an arrayed offset, where nine consecutive spectra were taken from the lactate region and the tenth spectrum was from the glucose region (spectral width 6 kHz and 512 points, shift of 8 kHz between glucose and lactate regions). For experiments with DHA, spectra were acquired with a nominal flip angle of 25°, TR of 1 s, spectral width 6 kHz, 1024 points. For chemical shift imaging with hyperpolarized [1-^13^C]DHA, 0.2 ml of the dissolution fluid was injected into the tail vein (19 s after the start of dissolution), and imaging started 15 s after injection. Chemical shift images of [1-^13^C]DHA and [1-^13^C]AA were acquired non-slice-selectively over the tumor region, using a 50-μs hard pulse, TR of 40 ms, echo time of 0.35 ms. Images were reconstructed in Matlab (MathWorks, Cambridge, UK). At 5 min after injection of hyperpolarized [1-^13^C]DHA, animals were sacrificed, and the tumors were excised and immediately freeze-clamped with liquid nitrogen-cooled tongs. Labeled DHA was extracted with 7% perchloric acid, and extracts were neutralized with KOH. Freeze-dried extracts were dissolved in 20 mm phosphate buffer, pH 7, containing 10% ^2^H_2_O and 10 mm ^13^C urea as a chemical shift standard. ^13^C spectra were acquired on a 600-MHz spectrometer (Bruker) at 300 K with a nominal flip angle of 30°, a TR of 3 s, and 12,000 transients. Peaks were integrated and normalized to the ^13^C urea standard using Topspin version 2.1 (Bruker).

### ^13^C Magnetic Resonance Spectroscopy Measurements on Cells in Vitro

Two ml of dissolution fluid containing hyperpolarized [1-^13^C]AA (14 mm) were injected into 2 ml of either water, RPMI medium, hydrogen peroxide solution, or an EL4 cell suspension in a 10-mm NMR tube. Spectra were acquired using a 9.4 T vertical wide bore magnet (Oxford Instruments) and a 10-mm broadband probe (Varian NMR Instruments, Palo Alto, CA). A total of 180 ^13^C spectra were acquired with an 8° flip angle pulse, TR of 500 ms, and spectral width of 16 kHz (18).

### Statistical Analysis

Statistical analyses were performed using Prism version 6 (GraphPad, San Diego, CA). Unpaired two-tailed *t* tests were used for all experiments, and significance was assumed at *p* < 0.05.

## Author Contributions

K. N. T. designed the study; performed, analyzed, and interpreted most of the data; and wrote the manuscript. D.-E. H. helped with the acquisition of all *in vivo* data and performed all procedures involving live mice. M. W. acquired and analyzed some of the data. A. J. W. helped with the acquisition and analysis of some of the data. M. I. K. helped with the acquisition and analysis of some of the data and commented on the manuscript. B. W. C. K., T. J. L., P. D., and I. M.-R. helped with the acquisition of some of the data and commented on the manuscript. S. E. B. designed the study, acquired and analyzed some of the data, and commented on the manuscript. K. M. B. designed the study, helped interpret the data, and wrote the manuscript.

## Footnotes

5 The abbreviations used are:

ROS: reactive oxygen species
PPP: pentose phosphate pathway
MRS: magnetic resonance spectroscopy
MRSI: MRS imaging
6PG: 6-phosphogluconate
3PG: 3-phosphoglycerate
DHA: dehydroascorbic acid
AA: ascorbic acid
Grx: glutaredoxin
TrxR: thioredoxin reductase
PMS: phenazine methosulfate
BSO: buthionine sulfoximine
G6PDH: glucose-6-phosphate dehydrogenase
PEP: phosphoenolpyruvate
STR: short tandem repeat.
